# Supplementary material for: Stacking Model‐Based Classifiers for Dealing With Multiple Sets of Noisy Labels
Source: Biom J. 2025 Mar 12;67(2):e70042. doi: 10.1002/bimj.70042 (PMC11898607; doi:10.1002/bimj.70042)
Supplement: Supplementary file 1 — Supporting Information [file BIMJ-67-e70042-s001.zip › density-based-ensemble-model-main/competitors_real_data/netlab-master/netlab-master/help/rbftrain.htm]

Netlab Reference Manual rbftrain


# rbftrain

## Purpose

Two stage training of RBF network.

## Description

`net = rbftrain(net, options, x, t)` uses a
two stage training
algorithm to set the weights in the RBF model structure `net`.
Each row of `x` corresponds to one
input vector and each row of `t` contains the corresponding target vector.
The centres are determined by fitting a Gaussian mixture model
with circular covariances using the EM algorithm through a call to
`rbfsetbf`. (The mixture model is
initialised using a small number of iterations of the K-means algorithm.)
If the activation functions are Gaussians, then the basis function widths
are then set to the maximum inter-centre squared distance.

For linear outputs,
the hidden to output
weights that give rise to the least squares solution
can then be determined using the pseudo-inverse. For neuroscale outputs,
the hidden to output weights are determined using the iterative shadow
targets algorithm.
Although this two stage
procedure may not give solutions with as low an error as using general
purpose non-linear optimisers, it is much faster.

The options vector may have two rows: if this is the case, then the second row
is passed to `rbfsetbf`, which allows the user to specify a different
number iterations for RBF and GMM training.
The optional parameters to `rbftrain` have the following interpretations.

`options(1)` is set to 1 to display error values during EM training.

`options(2)` is a measure of the precision required for the value
of the weights `w` at the solution.

`options(3)` is a measure of the precision required of the objective
function at the solution. Both this and the previous condition must be
satisfied for termination.

`options(5)` is set to 1 if the basis functions parameters should remain
unchanged; default 0.

`options(6)` is set to 1 if the output layer weights should be should
set using PCA. This is only relevant for Neuroscale outputs; default 0.

`options(14)` is the maximum number of iterations for the shadow
targets algorithm;
default 100.

## Example

The following example creates an RBF network and then trains it:

```
net = rbf(1, 4, 1, 'gaussian');
options(1, :) = foptions;
options(2, :) = foptions;
options(2, 14) = 10;  % 10 iterations of EM
options(2, 5)  = 1;   % Check for covariance collapse in EM
net = rbftrain(net, options, x, t);
```

## See Also

`rbf`, `rbferr`, `rbffwd`, `rbfgrad`, `rbfpak`, `rbfunpak`, `rbfsetbf`

---

**Pages:**
Index


---

Copyright (c) Ian T Nabney (1996-9)
